# Down-Regulation of *miR-200c* and Up-Regulation of *miR-30c* Target both Stemness and Metastasis Genes in Breast Cancer 

**DOI:** 10.22074/cellj.2020.6406

**Published:** 2019-07-31

**Authors:** Mahsa Rahimi, Ali Sharifi-Zarchi, Nosratollah Zarghami, Lobat Geranpayeh, Marzieh Ebrahimi, Effat Alizadeh

**Affiliations:** 1 Department of Medical Biotechnology, Faculty of Advanced Medical Sciences, Tabriz University of Medical Sciences, Tabriz, Iran; 2 Department of Stem Cells and Developmental Biology, Cell Science Research Center, Royan Institute for Stem Cell Biology and Technology, ACECR, Tehran, Iran; 3 Department of Surgery, Sina Hospital, Tehran University of Medical Sciences, Tehran, Iran; 4 Drug Applied Research Center, Tabriz University of Medical Sciences, Tabriz, Iran

**Keywords:** Metastasis, *miR-200c*, *miR-30c*, Self-Renewal, Spheroid

## Abstract

**Objective:**

microRNAs (miRNAs) play important role in progression of tumorigenesis. They can target self-renewal and epithelial-mesenchymal transition (EMT) abilities in tumor cells, especially in cancer stem cells (CSCs). The objective of this study was to implement data mining to identify important miRNAs for targeting both self-renewal and EMT. We also aimed to evaluate these factors in mammospheres as model of breast cancer stem cells (BCSCs) and metastatic tumor tissues.

**Materials and Methods:**

In this experimental study, mammospheres were derived from MCF-7 cells and characterized for the CSCs properties. Then expression pattern of the selected miRNAs in spheroids were evaluated, using the breast tumor cells obtained from seven patients. Correlation of miRNAs with self-renewal and EMT candidate genes were assessed in mammospheres and metastatic tumors.

**Results:**

The results showed that mammospheres represented more colonogenic and spheroid formation potential than MCF-7 cells (P<0.05). Additionally, they had enhanced migration and invasive capabilities. Our computational analyses determined that *miR-200c* and *miR-30c* could be candidates for targeting both stemness and EMT pathways. Expression level of *miR-200c* was reduced, while *miR-30c* expression level was enhanced in mammospheres, similar to the breast tumor tissues isolated from three patients with grade II/III who received neo-adjuvant treatment. Expression level of putative stem cell markers (*OCT4, SOX2, c-MYC*) and EMT-related genes (*SNAIL1, CDH2, TWIST1/2*) were also significantly increased in mammospheres and three indicated patients (P<0.05).

**Conclusion:**

Simultaneous down-regulation and up-regulation of respectively *miR-200c* and *miR-30c* might be signature of BCSC enrichment in patients post neo-adjuvant therapy. Therefore, targeting both *miR-200c* and *miR-30c* could be useful for developing new therapeutic approaches, against BCSCs.

## Introduction

Breast cancer is the second-most prevalent cancer between females worldwide (1) and effective treatment of breast cancer is faced to a number of hurdles including resistance to therapies, metastasis and recurrence (2). There are several evidences regarding the heterogeneity of breast cancer cell population, initiated from a very slight subset of cells named cancer stem cells (CSCs) (3). CSCs with self-renewal capacity are responsible for initiation of tumorigenesis in immunodeficient models (4) as well as maintenance and clinical outcomes of treatments (5). Although CSCs play central role from clinical points of view, molecular mechanisms and pathways involved in their survival and maintenance has not fully been identified (6). Increasing our knowledge in the field of tumor biology could consequently lead to suggestion of effective diagnostic and prognostic methods, as well as more impressive treatment for breast cancer. Moreover, recent progress has highlighted the important role of microRNAs (miRNAs) in regulating stemness and metastasis of CSCs. In this way, several miRNAs are known to be differentially expressed in CSCs or normal stem cells, part of which has been studied in targeting genes and networks involved in cancer stemness properties (7). On the other hand, the regulatory role of miRNAs has been defined in epithelial-mesenchymal transition (EMT), as an important process through tumor progression (8). Although it is known that miRNAs could contribute to tumorigenesis as tumor suppressors or oncogenes (9, 10), the role of miRNAs targeting both self-renewal and EMT pathways in breast CSCs (BCSCs) has largely been remained unknown. 

Recently, with regards to the new technologies innovation, data mining and bioinformatics approaches have tremendously been developed in the field of genomic analysis large-scale endeavors created useful databases. We hypothesized that identification of common miRNAs targeting stemness-EMT network will improve our understanding of CSCs in metastatic breast cancer. To date, several investigations have been performed to find deregulated miRNA expression during EMT and metastasis of breast cancer or BCSCs. In this study, our approach is systematic analysis of combined clinical and molecular data to find common miRNAs deregulated in mammospheres, as BCSCs model, and metastatic breast cancer. To reach that, we integrated candidate miRNA expression profiles with their target mRNA gene expression data obtained from the same samples. In summary, our findings resulted to understand the important role of *miR-200c* and *miR-30c* in maintenance of stemness as well as EMT process in BCSCs. Therefore, we suggested that down- regulation of *miR-200c* combined with increasing level of *miR-30c* may be a signature of BCSCs enrichment in patients post neo-adjuvant therapy. These miRNAs may have potential to extent into both diagnostic filed, as biomarker, and therapeutic approach for BCSCs in patients who are under chemotherapy.

## Materials and Methods

In this experimental study, breast cancer tissues were collected between January 2017 and January 2018, upon the approval of Farmanieh Hospital and Sina Oncologic Hospital (both from Tehran, Iran) according to local authorities. All contributors signed a written informed consent form to participate in this study. All procedures performed in studies including human patient involvements were in accordance with the ethical standards that approved by Tabriz University of Medical Sciences (5/D/25333) and Royan Institute Ethical Committee (IR.ACECR.ROYAN.REC.1396.229), as well as the 1964 Helsinki declaration and its later amendments or comparable ethical standards. Patients histopathological information, including tumor size and depth of invasion, lymph-vascular and perineural invasion, grade and clinical tumor/node/metastasis, were recorded and pathologically staged using the tumor-nodes-metastasis (TNM) staging method (11). Informed consent was obtained from all participants included in the present study in Sina and Farmanieh hospitals, Tehran, Iran.

Seven female breast cancer patients who underwent surgery at Farmanieh Hospital and Sina Oncologic Hospital were included in this research. The inclusion criteria for selection of female patients were 25 years of age and older, from all ethnicity. Breast cancer malignancy was confirmed based on histopathological examination and immunohistochemical studies of estrogen receptor (ER) and progesterone receptor (PR) expressions, performed on surgical resection tissue samples of the tumors based on the standard methods. Three samples were undergoing neo-adjuvant therapy before sampling. Normal adjacent biopsies, as negative controls, were collected from all seven patients. For sampling, surgeon removed the tumors and small part of them was cut for cultivation, which transferred to phosphate buffer saline (PBS) containing penicillin/streptomycin and the reminding part of tissues were fixed for pathological evaluation. Adjacent breast tissues or the areas around tumor sites were removed and transferred to transferring media (PBS containing penicillin/streptomycin) in the separate tube. Later on, these samples are called as normal tissues in the present study. 

### Literature mining and computational analysis

First we performed a systematic literature review on PubMed and COREMINE website using the following keywords: "breast cancer tissue, stem cell, self-renewal, stemness, miRNA, metastasis or EMT". The studies with incomplete data were excluded from this analysis, providing that: i. The papers are review articles or letters, ii. Studies with insufficient or inaccessible data, and iii. Studies that are not related to CSCs and homo-sapiens. We also excluded nine articles, due to limitation to access to their full texts. Moreover, miRNA expression profiles were searched with the same keywords on NCBI GEO database. In overall, we found the most frequent miRNAs targeting the stemness and metastasis genes. Then, we used miRNA target prediction tools including TargetScan (12) and miRWalk (13), to find target genes of each candidate miRNA. We only preserved the target genes with at least two-fold expression change and P<0.05, between human breast cancer versus human normal breast (the first group) and mammosphere versus MCF-7 adherent culture (the second group). Custom R scripts were used to rank miRNAs for targeting at least three stemness and two metastasis genes. Subsequently, we computed differential expression fold-changes and P values (using two-sided Student’s t test) between breast cancers vs. normal breast (as the first group) and also mammospheres vs. MCF-7 adherent culture (as the second group). Enricher (14) and GO functional enrichment analysis on KEGG 2017 pathways were used to identify pathways and biological functions that were affected by the target genes of each miRNAs. 

### Cell line and monolayer culture

MCF-7 is an estrogen-dependent human breast adenocarcinoma cell line that was purchased from Iranian Biological Resource Center (IBRC), Iran. The cells were cultured in Dulbecco’s Modified Eagle Medium (DMEM, Gibco, USA) supplemented with 10% heat inactivated fetal bovine serum (FBS, Invitrogen, USA), 1% nonessential amino acid (NEAA) , 2 mM L-glutamine and 1% penicillin/streptomycin (all from Life Technologies, USA) at 37°C and 5% CO_2_ using standard cell culture incubator.

### Formation of spheroid cultures from MCF-7 

The standard tissue culture plates were covered with poly 2-hydroxyethyl methacrylate (poly-HEMA) preventing cell attachment to plate surface. Subsequently, the monolayer MCF-7 cells were enzymatically detached into single cells suspension with trypsin (Gibco, USA) and harvested. 2×10^4^ single cells were seeded at low attachment plate, in serum-free DMEM medium enriched with 20 ng/ml epidermal growth factor (EGF, Royan Institute, Iran), 20 ng/ml basic fibroblast growth factor (bFGF, Royan Institute, Iran), 2% B27 (Gibco, USA) and 2 mM L-Glutamine (Life Technologies, USA). The media was refreshed every 48 hours and mammospheres were formed after 14 days. 

### Mammosphere-forming efficiency assay

When the spheroids reached to about 50 µm diameters, they were accumulated by gentle centrifugation at 1000 rpm for 5 minutes, and then were enzymatically separated with trypsin. About 2×10^4^ cells were plated into poly- HEMA coated six-well plates in 2000 µl of serum-free DMEM medium per well. Mammosphere-forming efficiency (MFE) was calculated by dividing the number of mammospheres, which are greater than 60 µm or larger in size in the cells seeding density per well using a microscope fitted with magnitude. All experiments on each generation of mammospheres were performed in triplicates. 

### Colony-forming test

To compare colony forming capacity of the adherent cells and mammospheres, 200 cells of each group were counted and re-plated in a complete medium containing DMEM supplemented with 10% FBS, 1% NEAA, 2 mM L-glutamine and 1% penicillin/streptomycin in six-well-plates. After 10 days, cell colonies were fixed with 4% paraformaldehyde, and stained with 0.05% crystal violet (Sigma, USA). Ultimately, the round shape colonies with more than 400 µm diameter were counted using an inverted microscope (Japan Microscope brand, Japan). 

### Transmembrane migration and invasion assay

Adherent cells and mammospheres were grown up to 80% confluence. Then adherent cells were starved in serum-free medium the day before assay. The next day, the cells were dissociated into single cells with trypsin, counted and added at 1×10^5^ cells/well density onto the top chambers of trans-well inserts of 8 µm pore size filter (BD, USA) coated with 0.5 mg/ml Matrigel (BD, USA) in a six-well plate. DMEM containing 10% of FBS was added to the bottom of chambers and the cells were then cultured for 24 hours at 37°C in a 5% humidified CO_2_ incubator. Finally, the cells on the top surface of filter were removed from filter surface by using a cotton-swab, and cells at the bottom of filter were then fixed with 4% paraformaldehyde (Merk, Germany), stained with 0.05% crystal violet (Sigma, USA) for 30 minutes. Very carefully, to avoid washing off the fixed cells, the membrane was dipped into distilled water to remove the excess crystal violet. Trans-well membrane was next allowed to dry. 

The cells were observed using an inverted microscope with either ×4 or ×10 objective lens and number of the cells were quantified in different fields of view to get an average sum of cells invaded through the membrane and attached to the underside of membrane. For migration assay, all steps were carried out similar to those in the invasion assay, except the matrigel coating. All experiments were performed in triplicates. 

$$\mathrm{Determining percent of invasion}=\frac{\mathrm{Mean number of cells invading through matrigel matrix-coated membrane}}{\mathrm{Mean number of cells migrating through uncoated membrane}}\times 100$$

### Quantitative real time polymerase chain reaction analysis of gene expression

Tumor and normal breast tissue fragments (<3×3 mm) were snap frozen in liquid nitrogen and homogenized with a ceramic pestle in TRIzol Reagent (Invitrogen, USA). Total RNAs with the aim of small RNA retentions were extracted from the adherent cells (as control groups) and mammospheres (as experimental groups) using TRIzol reagent, according to the manufacturer’s instructions. The concentration and purity of extracted RNA were determined by UV absorbance at 260 and 280 nm (260/280 nm) in spectrophotometer. The integrity of RNA samples was checked by gel electrophoresis. 2 µg total RNAwas subjected to generate complementary DNA using cDNA synthesis kit (TaKaRa, Japan), according to the manufacturer’s instructions. Expression level of stemness and metastasis genes was evaluated by Applied Biosystems real-time PCR Instrument (ABI, Thermo Fisher, USA) in 10 µl reactions containing 2.5 µl SYBR Green PCR mix (TaKaRa, Japan) and 1 µl of each primer with 5 pmol/µl concentration. Specific human primers -including stemness related genes (*OCT4, SOX2, NANOG, c-MYC* and *KLF4*) and metastasis related genes (*CDH1, CDH2, SNAIL1, TWIST1, TWIST2* and *ZEB1*) were used (Table S1) (See Supplementary Online Information at www.celljournal.org). PCR program was incubated at 95°C for 10 minutes, 40 cycles of denaturation at 95°C for 10 seconds, annealing at 60°C for 20 seconds and elongation at 72°C for 20 seconds. A final melting curve analysis from 65°C to 95°C was performed and the relative levels of expression were analyzed using 2^-ΔΔCt^ values. *ß-Actin* was used as house-keeping gene. 

### miRNA expression profiling 

miRNA expression levels were studied by performing SYBR Green qRT-PCR. In brief, 1 µg total RNA containing miRNAs was poly adenylated by poly (A) polymerase and reverse transcribed to cDNAusing reverse transcriptase enzyme first strand cDNAsynthesis reaction, provided from Parsgenome miR-Amp kit (Parsgenome, Iran) according to the manufacturer’s instructions. Each reaction was performed in a final volume of 10 µl, containing diluted cDNA and PCR master mix, and all reactions were run in triplicates. qRT-PCR reaction was performed using Applied Biosystems Real-Time PCR Instruments according to the manufacturer’s protocol. Expression levels of miRNA were normalized against internal controls U6, as a housekeeping control. 

### Statistical analysis

*In vitro* characterization of MCF-7 cell mammosphere and primary breast cancer tissue are presented as the mean ± SD of at least three different experiments. Two-tailed Student’s t test and analysis of variance (ANOVA) were performed to evaluate the difference between the mean values. The Spearman’s rank correlation test was used to evaluate miRNAand mRNA correlation. A two-tailed analysis with P<0.05 was considered statistically significant for all experiments. For functional enrichment analysis, target genes of the selected miRNAs were submitted to Enrichr database. Subsequently, biological process, cellular component and molecular function were analyzed by Gene Ontology (GO) and pathways analysis was applied by KEGG 2017 (P<0.05).

## Results

### Computational analysis to identify common miRNA in stemness and EMT network

**Fig.1:**
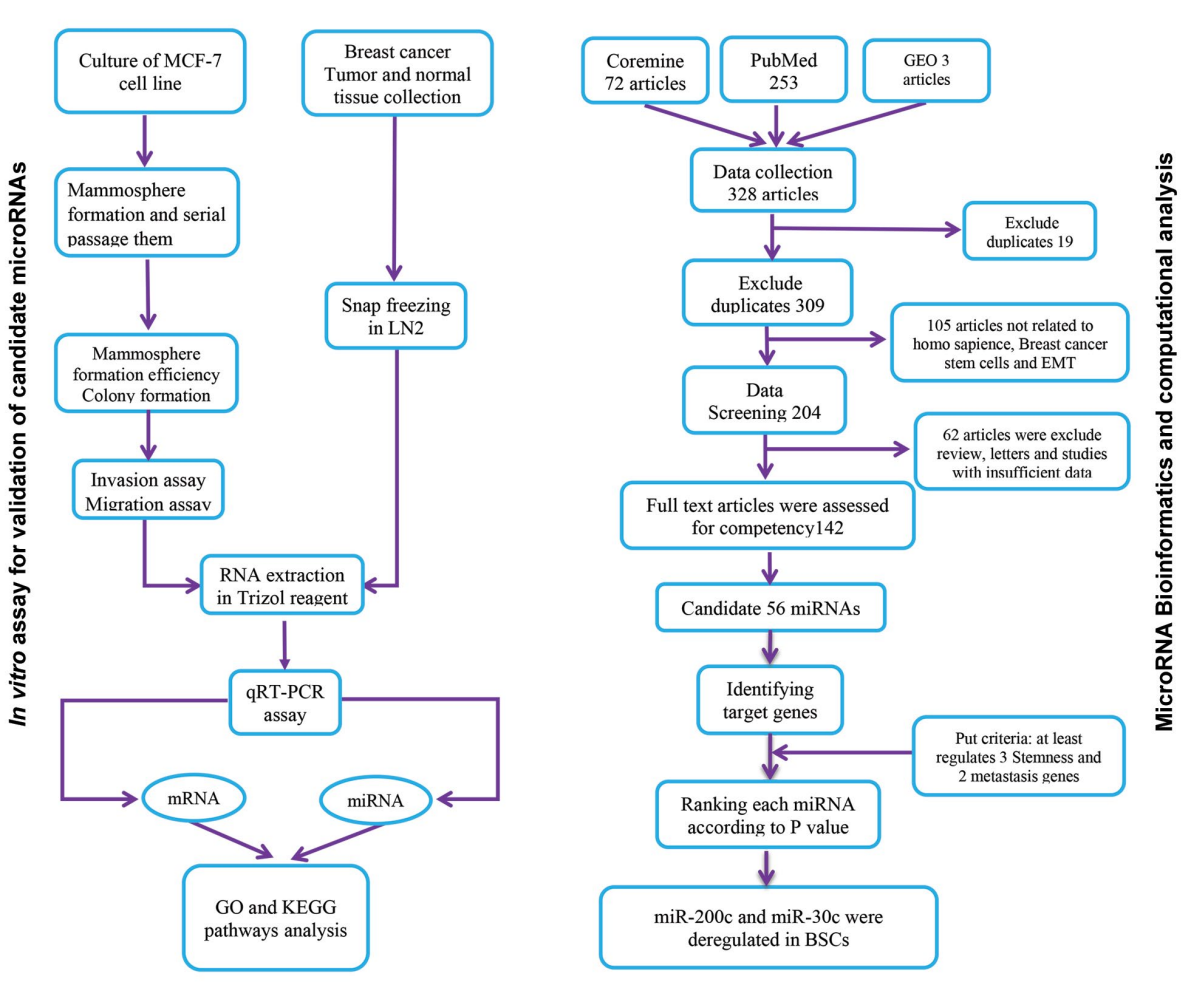
Flowchart of studies represent systematic analysis to find miRNAs targeting both self-renewal and EMT pathways. EMT; Epithelial-mesenchymal transition, qRT-PCR; Quantitative real time polymerase chain reaction, GO; Gene ontology, KEGG; Kyoto Encyclopedia of genes and genomes-genomenet, GEO; Gene expression omnibus, and BSCs; Breast cancer stem cells.

A total of 328 articles were yielded after the literature reviews, finally limited to 142 papers due to our exclusion criteria (mentioned in the method section). Full-text reviews were resulted in proposing 56 candidate miRNAs that have key role in BCSCs: 24 up-regulated and 32 down-regulated molecules. Among them, we chose *miR-200c* and *miR-30c* targeting at least three stemness and two EMT genes (Fig .1). 

### Mammospheres derived from MCF-7 as a model of breast cancer stem cells

**Fig.2:**
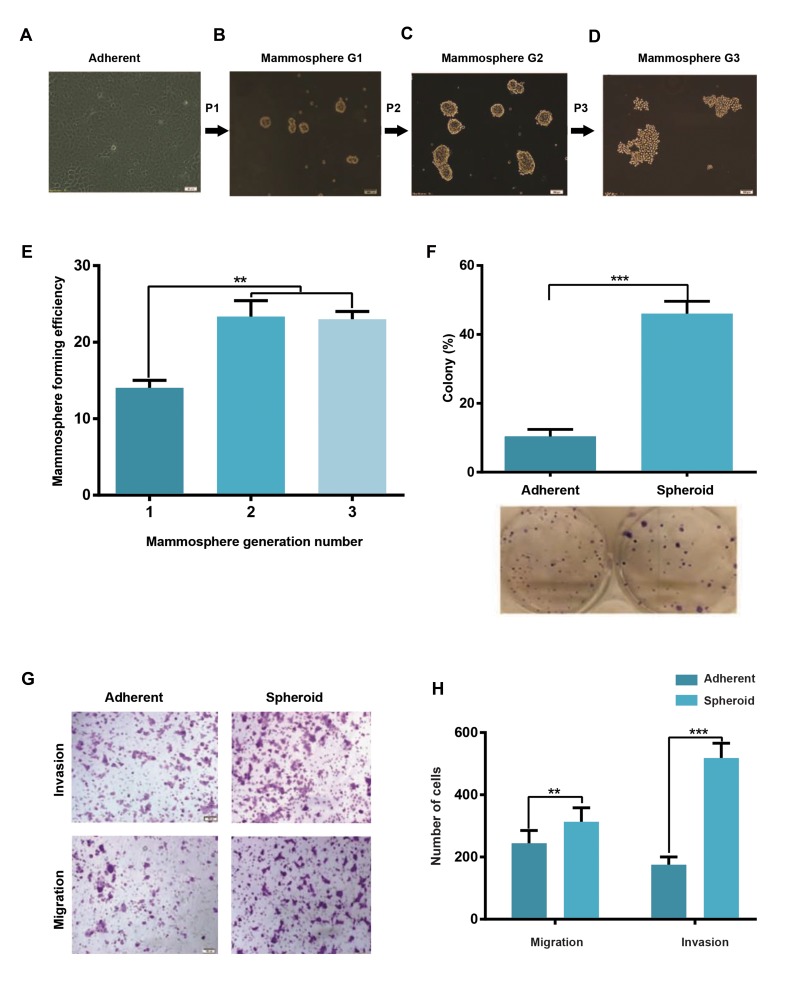
Colony and mammosphere formation abilities in MCF-7 and mammospheres. **A.** Parental cells cultured in 2D monolayer condition (magnification: ×4, scale bar: 100 µm), **B-D.** Serial mammospheres derived from the first generation of mammospheres (up to passage 3) showing progressive loss of cell cohesion and formed loos spheroid (magnification: ×20, scale bar: 100 µm), **E.** Mammosphere forming efficacy (MFE) was calculated from the first to third generation. Data are based on the mean percentages of the formed spheres quantity within a culture relative to the initial cell seeding number (mean ± SD, n=3), **F.** The percentage of colonies increased in cells derived from mammospheres in compare to the adherent cells. (mean± SD, n=3), **G.** Evaluation of migration and invasion abilities of the cells isolated from mammospheres and MCF-7 monolayer. Left panel shows crystal violet stained cells, passing through the matrigel coated filter insert (as invasive cells) or uncoated filter insert (as migratory cells), and **H.** Quantification of migratory and invasive cells in adherent vs. mammosphere cells. Mammospheres revealed higher migration and invasion rate than their parental cells. Bars indicated mean ± SD of three independent experiments. **; P<0.01 and ***; P<0.001.

MCF-7 cells were grown similar to adherent epithelial- like monolayer cells in culture (Fig .2A). Under serum- free and low-attachment conditions, MCF-7 cells grew into 3D non-linkage mammospheres within 24-48 hours, in comparison with their 2D adherent culture. Shape and appearance of spheres are solid and tightly packed in rounded margin, but we observed the mammospheres form looser and less rounded spheres over passages (Fig .2B-D). The secondary spheres were subsequently cultured up to three passages and MFE was calculated based on their size (> 60 µm, Fig .2E). The spheroid cells indicated about two folds increase (P<0.05) in MFE during three passages (Fig .2E). The results showed that MCF-7 cell-derived mammospheres had more colonogenic potential up to 5.5 fold. Indeed, the number as well as size of colonies was dominantly increased in mammospheres, in comparison with adherent cells (P<0.05, Fig .2F). 

### Mammospheres revealed increased ability of migration and invasion

The migratory capability of mammospheres was increased (about 1.7 fold). However, invasiveness potential of the isolated cells from mammospheres was significantly increased (about 3.35 fold), in comparison with adherent cells (Fig .2G, H).

### Patients’ demography

Seven female breast cancer patients (mean age of 48 ± 8.04 years) were included in the study after signing written informed consent. The clinicopathological data of all patients has been shown in Table 1. All tumors were classified as invasive ductal carcinoma (IDC). Immunohistochemical study of ER and PR expressions were performed on surgical resection tissue samples of the tumors based on the standard methods. Three samples were positive for ER, PR and HER2 and four patients were undergoing neo-adjuvant therapy before sampling. 

### Expression of *miR-200c*-3p and *miR-30c*-5p in tumor/ normal tissues and mammospheres/adherent cells

**Fig.3:**
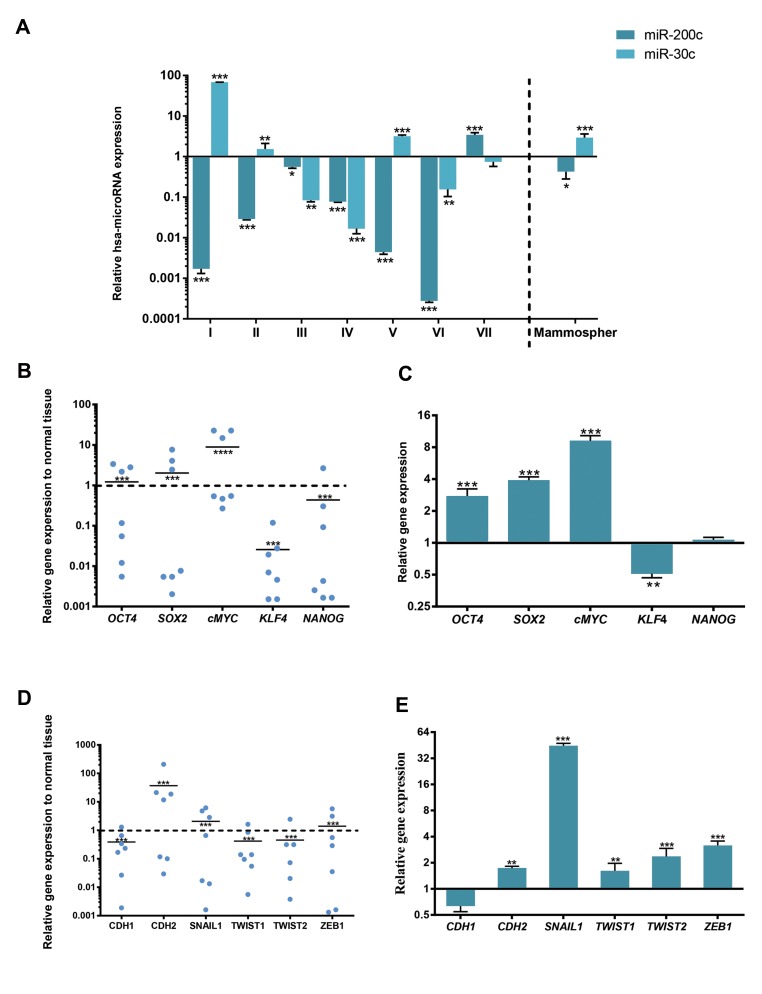
Expression level of miRNAs and genes. Expression levels of *miR-200c* and *miR-30c* as well as stemness and metastasis genes in human breast cancer versus normal breast (the first group), and mammospheres versus MCF-7 adherent cells (the second group) were determined by quantitive real time polymerase chain reaction (qRT-PCR). **A.** Expression of each miRNA was normalized to the levels of U6. Each cell line represents n=3 and tumor represents n=1. **B, C.** Scatter plot of stemness and metastasis gene expression levels in breast cancer and normal breast tissues (control). The line represents mean value, **D,** and **E.** Expression level of stemness and metastasis genes in mammospheres related to adherent cells (control), determined by qRT-PCR. *ß-Actin* was used as the housekeeping gene. Statistically significant difference was determined by paired t test with GraphPad Prism 6 software. Bars indicated mean ± SEM.

Findings showed that *miR-200c* was decreased in mammospheres, compared to parental MCF-7 cells (P=0.0025, Fig .3A, Right). Furthermore, this expression was down-regulated in breast cancers with metastatic conditions (patients I, II and V, Fig .3A, Left). In contrast, expression of *miR-30c* was overexpressed in mammospheres, compared to adherent cells (P=0.0011), and it was also up-regulated in three of patients with grade II/III who received neo-adjuvant therapy (Fig .3A). 

### Gene expression in tumor/normal tissues and mammospheres/adherent cells

In the next step, expression level of stemness related genes (*OCT4, SOX2, KLF4, c-MYC* and *NANOG*) and EMT transcription factors (*CDH1, CDH2, SNAIL1, TWIST1, TWIST2* and *ZEB1*) were evaluated in all tumor samples and mammospheres. Interestingly, expression level of *OCT4, SOX2* and *c-MYC* was significantly increased in mammospheres and three malignant breast tumors who were under neoadjuvant therapy (patients I, II and V, Fig .3B, C). *KLF4* was down-regulated in both tumor samples and mammospheres. Meanwhile, expression of *NANOG* was not changed in mammospheres, but it was down-regulated in tumors (Fig .3B). Among EMT-related genes, *CDH2, SNAIL1, TWIST1/2* and *ZEB1* were also overexpressed in mammospheres. However, tumors differentially expressed EMT related genes. Expression of *CDH2, SNIL1* and *ZEB1* were up-regulated in three malignant breast tumors (patients I, II and V), but the others were down- regulated (Fig .3D, E). This demonstrates that signature of self-renewal related gene expressions and some EMT genes in malignant breast tumor of patients who underwent neo-adjuvant therapy are similar to that of mammospheres, as BCSC model.

**Table 1:** Clinicopathological features of breast cancer patients


Patients	Age (Y)	Histological subtype	Ki-67	Grade	ER status	PR status	HER2 status	Metastasis	Neo-adjuvant

Case I	56	IDC	>10%	III	>80%	>80%	Negative	Yes	Yes
Case II	51	IDC	>30%	III	60%	20%	30%	Yes	Yes
Case III	45	IDC	NA	I	NA	NA	NA	No	Yes
Case IV	50	IDC	NA	NA	NA	NA	NA	No	NA
Case V	39	IDC	>50%	II	>80%	>80%	>30%	Yes	Yes
Case VI	58	IDC	NA	I	NA	NA	NA	No	No
Case VII	37	IDC	NA	I	NA	NA	NA	No	No


IDC; Invasive ductal carcinoma, ER; Estrogen receptor, PR; Progesterone receptor, and NA; Not available.

### Correlation of *miR-200c* and *miR-30c* with stemness and EMT genes, and with overall survival of breast invasive carcinoma 

In next step, correlation of *miR-200c* and *miR-30c* with stemness and metastasis gene expressions were assessed in both mammospheres and tumor tissues. As shown in Table 2, *miR-200c* was negatively correlated to *SOX2* and *KLF4* stemness genes, as well as *SNAIL* and *TWIST1* EMT genes. However, expression of *miR-30c* strongly displayed positive correlation with expression of most stemness related gens “*OCT4, SOX2, KLF4* and *NANOG*” and all EMT related genes. Moreover, *miR-200c* had negative correlation with *miR-30c* (R=-0.8, P=0.04). All aforementioned data indicates discriminatory potential of *miR-30c* and *miR-200c* to target both EMT and self- renewal pathways in BCSCs and malignant breast tumors. 

### Target genes and pathways analyses for *miR-200c*-3p and *miR-30c*-5p 

**Fig.4:**
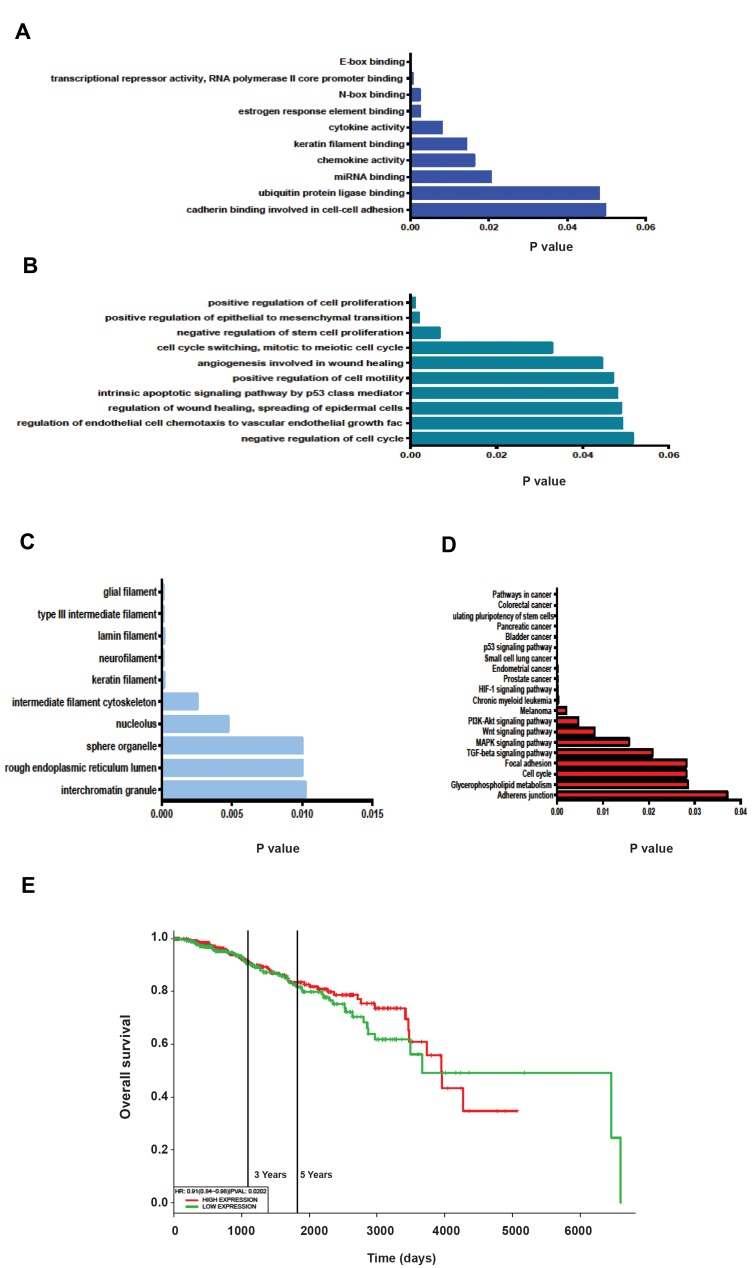
Gene ontology (GO), KEGG Pathway analysis using Enrichr and diagnostic plots creation with PROGmiR. **A.** Molecular function of stemness and epithelial-mesenchymal transition (EMT) regulated genes through the differentially expressed miRNAs, **B.** Biological process, **C.** Cellular component of these genes. Only the top ten enriched GO terms are represented, **D.** KEGG pathways with their P values. The most significant pathways bear the smallest P value listed from top to down, and **E.** Kaplan-Meier survival curve analysis was associated with overall survival in patients’ breast invasive carcinoma cells. The patients were stratified into high-risk and low-risk groups according to median of each miRNA.

In order to recognize the potential miRNA efficacy for breast cancer tracing, we predicted target genes of *miR-200c*-3p and *miR-30c*-5p. They were listed to GO annotation dataset for analysis of molecular function, biological processes and cellular component by Enricher. The result was sorted based on p-value. The lowest P value is related to more specific term. GO analysis showed that targeted genes of the differentially expressed miRNAs were enriched in the molecular functions of E-box binding, DNA binding, N-box binding, estrogen response element binding, cadherin binding involved in cell-cell adhesion and miRNA binding (Fig .4A). The biological process of these genes included cell-cell adhesion, stem cell proliferation process, cell cycle, angiogenesis and EMT process (Fig .4B). In terms of cellular component, most of the genes belong to the nucleolus and cytoplasmic organelles (Fig .4C). Finally, KEGG pathway analysis also showed similar results, in terms of the number of genes involved in the adhesion junction, pathways in cancer, MAPK signaling pathway, Wnt signaling pathway, PI3KAKT signaling pathway, HIF-1 signaling pathway, TGF- beta signaling pathway, as well as the signaling pathways regulating pluripotency of stem cells, P53 signaling pathway and cell cycle (Fig .4D). Additionally, using PROGmiR (14), we were able to create a significant diagnostic plot between the expression level of individual *miR-200c* and *miR-30c*, and overall survival rate of the patients. Actually, simultaneous deregulation of miR200c and *miR-30c* could significantly reduce the survival rate of breast invasive carcinoma cells via up-regulation of *OCT4, SOX2, c-MYC, SNAI1, ZEB1, CDH2* and down- regulation of CDH1 (P=0.02, Fig .4E). 

**Table 2:** Spearman’s rho for stemness and epithelial-mesenchymal transition (EMT) genes


miR name	OCT4	SOX2	c-MYC	KLF4	NANOG	CDH1	CDH2	SNAIL1	TWIST1	TWIST2	ZEB1

miR-200c	R=-0.4	R=-0.94^*^^*^^*^	R=-0.5	R=-0.93^*^^*^^*^	R=-0.4	R=0.9^*^^*^	R=-0.4	R=-0.6^*^^*^	R=-0.8^*^^*^	R=-0.4	R=0.5
	P=0.4	P=0.005	P=0.08	P=0.0006	P=0.39	P=0.01	P=0.39	P=0.01	P=0.01	P=0.32	P=0.28
miR-30c	R=0.85^*^^*^^*^	R=0.95^*^^*^^*^	R=-0.3	R=0.71^*^^*^	R=0.9^*^^*^^*^	R=0.5^*^	R=0.74^*^^*^^*^	R=0.87^*^^*^^*^	R=0.92^*^^*^^*^	R=0.82^*^^*^^*^	R=0.83^*^^*^^*^
	P=0.000	P=0.003	P=0.29	P=0.004	P<0.001	P=0.04	P=0.002	P=0.001	P=2.9E-06	P=0.0002	P=0.0001


*; P<0.05, **; P<0.01, and ***; P<0.001.

## Discussion

This study evaluates expression of miRNAs targeting both stemness and metastasis pathways in BCSCs. To provide BCSC model, we used MCF-7 derived mammospheres representing higher ability to sphere and colony formations, compared to their parental cells, as well as more invasion and migration capabilities. Our data are in agreement with the previous studies (15, 16) that observed MCF-7 derived mammospheres contain highest CSCs population (17, 18) with CD44^+^/CD24^-^phenotype (19, 20). Although, we did not evaluate tumorigenicity of the mammospheres *in vivo*, but adequate studies have determined tumorigenic ability of the cells originated from MCF-7-mammospheres in less than 1000 cell per injection (21).

To specify miRNAs involved in both stemness and metastasis regulation, systematic analysis was done using the important genes contributed to both pathways. The results implicated that has-*miR-200c*-3p and has-*miR- 30c-5p* could potentially regulate these pathways. These miRNAs have been identified in different studies to control major transcription factors of EMT and induce metastasis (22, 23). It has also been reported that *miR-200c* controls BCSC functions (24, 25). Meanwhile, few studies implicated the role of *miR-30c* in BCSCs. Interestingly, the mammospheres of this study had similar expression pattern of *miR-200c* and *miR-30c* to three patients (I, II and V); these patients were at grade III/II and received neo-adjuvant therapy before sample collection. They showed significant down-regulation of *miR-200c* and up-regulation of *miR-30c*; however, *miR-200c* and *miR-30c* expression were both down- regulated in patients number six and four, among seven patients. Thus, by considering this similarity, we figured out the expression level of stemness related genes in mammospheres as well as all tissue samples. Impressively, expression of *OCT4, SOX2* and *c-MYC* was up-regulated in mammospheres and the same three previous patients (I, II and IV). *KLF4* expression, as another stemness related gene, was diminished in mammospheres and most of the tumor tissues, and *NANOG* was just significantly down- regulated in patient samples, but not in mammospheres. Moreover, transcription of *miR-30c* displayed positive correlation with *OCT4, SOX2, KLF4* and *NANOG* expressions. In addition, *miR-200c* had negative correlation with expression of *SOX2* and *KLF4*. Indeed, *miR-200c* significantly exhibited negative correlation with *miR-30c*. Similar to our data, *miR-200c* clusters (*miR-200c-141, miR-200b-200a-429* and *miR-183-96-182*) have been reported to be down-regulated in isolated BCSCs from eleven human breast cancers tissues, normal mammary stem cells (26, 27) and carcinoma cells (28). Furthermore, lower expression of *miR-200c* in patients could be considered as a prognostic factor of breast cancer metastasis, since its down-regulation associates with poor survival rate. Up-regulation of this miRNA correlates with inhibition of cell proliferation and regulates cancer stem cell functions (29-31). In addition, *miR-200c* plays an important role in inhibiting proliferation of breast cancer cells by targeting the stemness related genes such as *NANOG, SOX2* and *KLF4* that are located in down-stream of *miR-200c*. It also inhibits tumor growth, differentiation and self-replication of CSCs by targeting TUBB3 and as a result it would be involved in restoring sensitivity to microtubule-targeting drugs (32). 

In this study, *miR-30c* represents stronger correlation with most of the stemness related genes. This miRNA has previously been reported as a breast cancer prognostic biomarker (33) and its expression is various among different breast cancer subtypes. Higher *miR-30c* expression level was reported in luminal-A tumors and low miR30c expression level was observed in basal-like tumors (34). In fact, few evidences are available representing effect of *miR-30c* in regulation of stemness, with mainly focus on EMT regulation. In one study, Yu et al. (35) showed down-regulation of miR-30 family, exclusively *miR-30e*, interferes with tumor beginning BCSCs (in mammospheres as well as primary BCSCs acquired from breast cancer patients) through up-regulation of ubiquitinconjugating enzyme 9 (*Ubc9*) and integrin b3 (*ITGB3*). This up-regulation results in reduced self-renewal and anti-apoptotic features of BCSCs. Overexpression of *miR-30a* considerably decreased the sphere creation capability of MCF-7 cells, while deterrence of miR-30a intensely enhanced the number of mammospheres in the human breast cancer cell line, MCF-7(36). 

Consistent with other studies, the present study demonstrated correlation of *miR-200c* and *miR-30c* with expression of important EMT transcription factors (*SNAI, TWIST* and *ZEB1*), in tumors and mammospheres. miR200c not only is a malignancy biomarker, but also promote metastasis in poor metastatic cells in vivo, presence of which in serum of metastatic breast cancer patients can be indicated for brain metastases (37). *miR-200c* maintains cells in an epithelial state condition , via the regulation of mesenchymal genes such as *CDH2, SNAI1, SNAI2, TWIST1, TWIST2* and *ZEB1* (27). *miR-30c* contributes to miRNA-cytoskeleton regulation network and its target genes (i.e. *VIM, TWF1,* and *IL-11*) represent invasion, EMT and chemo-resistance molecular mechanisms (35). In treatment of breast cancer cells, *miR-200c* was also reported to induce apoptosis (38) and sensitize the cells to chemotherapy, radiotherapy and trastuzumab using therapy. Down-regulation of this molecule is known as marker for drug resistance in female genital tumors, such as ovarian, cervical and breast cancers (39). 

We further employed bioinformatics tools to find out the target genes and pathways of *miR-200c* and *miR-30c* coordinating stemness and metastasis. Pathway analysis indicated that these genes considerably associate with "adherens junction pathway", "pathways involved in cancer", "MAPK signaling pathway", "Wnt signaling pathway", "PI3K-Akt signaling pathway", "regulating pluripotency of stem cells", "P53 signaling pathway", "TGFß signaling pathway" and "HIF-1 signaling". All of these pathways have been reported to be related to several cellular activities including proliferation, migration, invasion, cell cycle, regulation of ER signaling in cancer and CSCs. Adherens junction pathways are also of the major mechanisms presented in stem cells, where raising documents have illustrated that remodeling of the cytoskeletal proteins could characterize stem cell destiny (40). To the best of our knowledge, this is the first experiment comparing mammospheres as BCSCs model with signature pattern of metastatic patients (pre or post neo-adjuvant therapy). Because of similarity of *miR-200c* and *miR-30c* expression levels in mammospheres and some of our patients (I, II and V), we suggest that combination of these miRNAs might predict outcome of adjuvant therapy or metastasis in patients. Down- regulation of *miR-200c* and up-regulation of *miR-30c* suggest that metastatic breast tumors and mammospheres are similar and they contribute to communal molecular mechanisms regulating stem cell functions such as self- renewal, proliferation, EMT and resistance to drug.

## Conclusion

The present study demonstrates that down-regulation of *miR-200c* and up-regulation of *miR-30c* promote BCSC features toward malignant breast tumors, leading to their resistance to neo-adjuvant therapy. These findings suggest a signature to predict metastasis post chemotherapy in breast cancer patients. However, further experiments are required in this regard. 
